# Radiation-Induced Sphenoid Wing Meningioma After Childhood Medulloblastoma: A Case Report

**DOI:** 10.7759/cureus.105217

**Published:** 2026-03-14

**Authors:** Christopher J Walker, Una Della Pietra

**Affiliations:** 1 Osteopathic Medical School, Nova Southeastern University Dr. Kiran C. Patel College of Osteopathic Medicine, Clearwater, USA; 2 Internal Medicine, Broward Health Medical Center, Fort Lauderdale, USA

**Keywords:** medulloblastoma, meningioma, orbital extension, pediatric cranial radiotherapy, sinonasal invasion, skull base tumor, sphenoid wing meningioma

## Abstract

Radiation-induced meningiomas (RIMs) are a potential late sequela of pediatric cranial radiotherapy and may arise after a prolonged latency, often 10-30+ years after exposure. We report a 28-year-old male patient treated for medulloblastoma with surgical resection and cranial radiotherapy at age three, who later underwent resection of a right sphenoid wing meningioma at age 16. Following a period of stability, surveillance imaging revealed progressive regrowth at the prior craniotomy site, extending into the superior right orbit. He subsequently presented with dizziness, headaches, and visual changes. MRI demonstrated multifocal enhancing lesions suspicious for radiographic recurrence, involving the anterior right frontal convexity, frontal sinus and ethmoid air cells, and superior right orbit, with mass effect on the orbital structures causing proptosis, as well as an additional right parietal calvarial lesion with adjacent dural thickening and enhancement concerning for a separate meningioma. His admission was further complicated by an acute right posterior cerebral artery infarct, which delayed operative management. This case highlights the complexity of early-latency, multifocal RIM with orbital and sinonasal extension and underscores the need for individualized long-term surveillance in patients treated with cranial radiotherapy during childhood.

## Introduction

Radiation-induced meningiomas (RIMs) are a well-recognized late complication of cranial radiotherapy in survivors of childhood and adolescent cancers. As long-term outcomes improve, they have become an increasingly important survivorship issue. Large survivor cohorts and pooled analyses consistently demonstrate that subsequent meningioma risk rises with increasing cranial radiation exposure, supporting a dose-dependent relationship, and that risk persists for decades after treatment [1-4]. Because many pediatric malignancies require central nervous system-directed radiotherapy, a substantial number of survivors remain at lifelong risk for secondary intracranial neoplasms, including meningiomas [5-7]. Medulloblastoma survivors are a particularly relevant subgroup, given the historical use of craniospinal irradiation with boost fields during early developmental periods, which can have meaningful long-term intracranial sequelae [8,9].

Compared with sporadic meningiomas, RIMs have been reported to present at younger ages and to exhibit clinically important differences in behavior, including a tendency toward multifocal disease, higher-grade histology in some series, and increased recurrence or progression risk after treatment or during active monitoring [10-15]. Latency from irradiation to meningioma diagnosis is often measured in decades, with many cohorts reporting values in the 20-30-year range; however, substantial variability exists, and shorter latency can occur, particularly in patients irradiated at very young ages [3,7,12,16,17]. These features complicate patient counseling and long-term management, particularly when multiple lesions arise over time or when progression occurs between surveillance imaging intervals [7,13].

Skull base meningiomas pose additional challenges due to their proximity to the optic apparatus, cavernous sinus, and other critical neurovascular structures. Orbital or paranasal sinus extension can also cause compressive symptoms and limit surgical corridors. While many studies describe RIM epidemiology and outcomes in broad terms, location-specific reporting for skull base lesions remains inconsistent across the survivorship literature. This gap is particularly evident for sphenoid wing tumors with orbital and sinonasal involvement, which limits practical guidance on operative feasibility, recurrence risk, and follow-up strategy in this subgroup [10,12,13]. Surveillance recommendations also remain variable, and systematic reviews have noted that evidence is insufficient to define a single standardized screening approach across all survivor populations [6,16,18].

We report a 28-year-old male patient with childhood medulloblastoma treated with cranial irradiation at age three, who developed a right sphenoid wing meningioma that was resected at age 16. He later presented with multifocal recurrent disease extending into the orbit and paranasal sinuses, with proptosis and visual symptoms. Imaging also suggested an additional meningioma. We contextualize this case within the existing literature on RIMs, with emphasis on latency, multiplicity, recurrence risk, and long-term surveillance considerations relevant to skull base disease.

## Case presentation

We present a 28-year-old male patient with childhood medulloblastoma treated with surgical resection and cranial irradiation at age three. At age 16, he underwent a right frontal craniotomy for resection of a right sphenoid wing meningioma involving the anterior skull base. Postoperatively, he developed recurrent seizures, and imaging demonstrated postsurgical right frontal encephalomalacia. His seizures were subsequently controlled for several years with levetiracetam and divalproex sodium.

**Figure 1:**
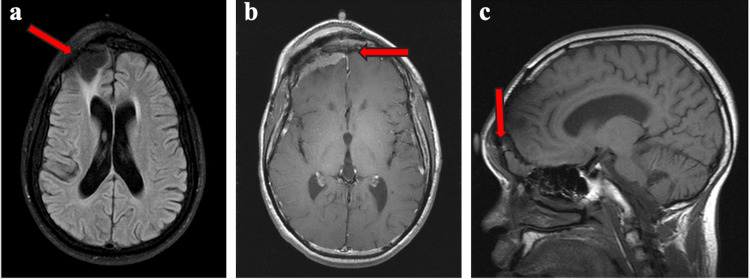
Brain MRI with and without contrast (August 2022). The red arrows indicate key imaging findings: (a) an axial T2 FLAIR image demonstrating diffuse cerebral volume loss with postsurgical right frontal encephalomalacia; (b) an axial post-contrast T1-weighted image showing a thick, irregular right frontal extra-axial enhancing rind consistent with recurrent tumor; and (c) a 0.9-cm extra-axial enhancing lesion adjacent to the right frontal lobe extending through the craniotomy defect toward the superior orbit.

At age 25, he noted gradual enlargement of a right frontal mass at the prior surgical site. MRI of the brain with and without contrast (August 2022) demonstrated new irregular right frontal extra-axial enhancement concerning for recurrent meningioma, with suspected trans-osseous extension through the prior craniotomy defect and associated postsurgical right frontal encephalomalacia (Figure 1). Because definitive management was deferred at that time, he was followed with serial imaging.

**Figure 2:**
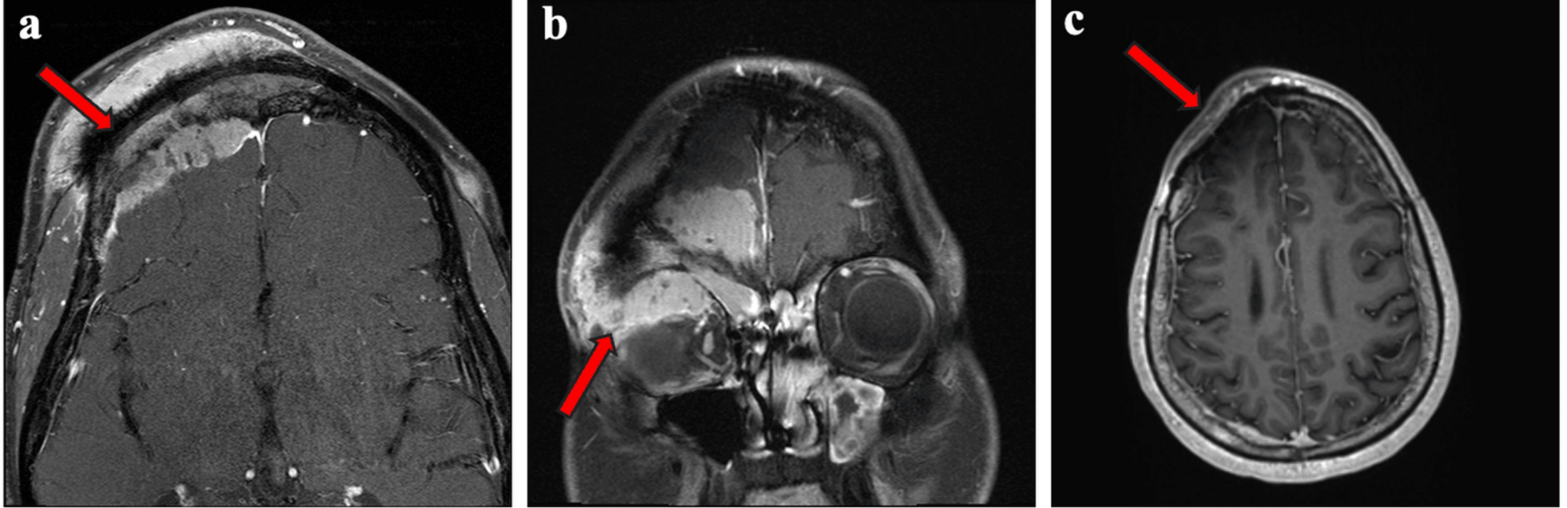
Post-contrast T1-weighted brain MRI (October 2024). The red arrows indicate enhancing lesions: (a) an avidly enhancing 3.4 × 7.0 × 4.7 cm transcalvarial right frontal mass extending from the scalp through the frontal bone/frontal sinus into the right frontal epidural space; (b) a 1.4-cm-thick enhancing mass in the superior right orbit centered at the prior right frontal/supraorbital cranioplasty; and (c) an enhancing right parietal calvarial lesion (~3.6 cm) with adjacent dural thickening/enhancement (up to 0.5 cm).

On follow-up MRI in October 2024, interval progression was demonstrated, including a large avidly enhancing transcalvarial right frontal mass extending from the scalp through the frontal bone, traversing the frontal sinus, and entering the right frontal epidural space. Additional findings included an enhancing superior right orbital mass centered at the prior right frontal-supraorbital cranioplasty and a separate enhancing right parietal calvarial lesion with adjacent dural thickening and enhancement (Figure 2). At that time, repeat surgical evaluation was recommended, but definitive intervention was deferred.

**Figure 3:**
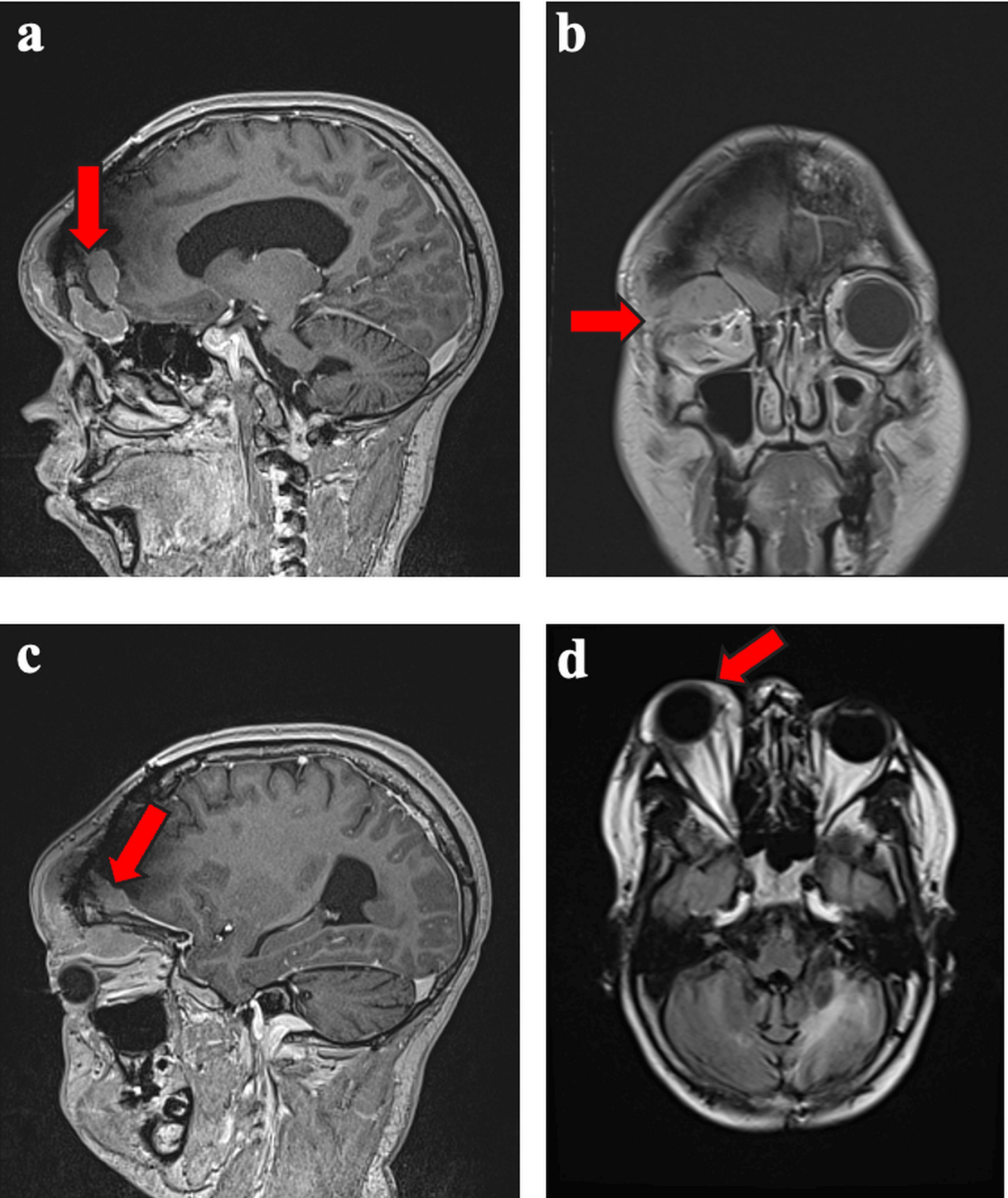
Post-contrast T1-weighted MRI brain (August 2025). (a) The arrow indicates a lobulated, enhancing extra-axial mass deep to the prior right frontal craniotomy along the anterior right frontal convexity, measuring 4.2 × 1.3 cm (sagittal). (b) The arrow demonstrates contiguous extension into the superior right extraconal orbit and frontal sinus/ethmoid air cells, measuring 4.2 × 1.5 cm (orbital component) and 3.2 × 1.2 cm (frontal sinus/ethmoid component), with associated right-sided proptosis (coronal). (c) The arrow demonstrates anterior extension toward the orbit and paranasal sinuses through the prior surgical site (sagittal). (d) The arrow highlights orbital involvement with mass effect on the right orbital structures consistent with recurrent disease (axial).

In August 2025, approximately 3 years after radiographic recurrence was first identified and 10 months after interval progression was documented, he presented with several days of dizziness, headaches, and visual changes. Examination revealed a right frontal mass with right eye proptosis. Brain MRI with and without contrast (August 2025) showed recurrent enhancing tumor along the anterior right frontal convexity deep to the prior craniotomy (4.2 × 1.3 cm), with contiguous extension into the frontal sinus and ethmoid air cells (3.2 × 1.2 cm) and into the superior right extraconal orbit and periorbital soft tissues (4.2 × 1.5 cm), causing mass effect on the right orbital structures and proptosis (Figure 3). A separate enhancing right parietal calvarial lesion with adjacent dural thickening and enhancement again raised concern for an additional meningioma. No residual or recurrent disease was identified at the prior posterior fossa medulloblastoma resection site.

During the admission, MRI also demonstrated an acute right posterior cerebral artery territory infarct involving the right occipital lobe with extension into the posterior mesial right temporal lobe, without hemorrhagic transformation. As part of the inpatient stroke evaluation, carotid duplex ultrasonography showed no hemodynamically significant extracranial carotid stenosis. No CT angiography or digital subtraction angiography was performed, and the stroke etiology remained undetermined in the available records. Neurosurgery recommended delaying operative intervention until neurologic stabilization and outpatient neurology follow-up, although definitive management remained deferred.

## Discussion

RIMs are increasingly recognized among survivors of childhood cancers treated with cranial radiotherapy and appear to differ clinically from sporadic meningiomas. Across published cohorts, multiplicity is commonly reported (approximately 11.9%-55.6%), and several series describe higher proportions of atypical histology and higher recurrence rates than sporadic disease in direct or indirect comparisons [10-13]. However, the original pathology report and WHO grade for our patient’s previously resected meningioma were not available, limiting direct comparison with these reported histologic patterns. These patterns have practical implications for counseling and long-term management because multifocal disease and recurrence can necessitate repeat interventions and prolonged surveillance. In the present case, imaging demonstrated recurrent disease with extensive skull base involvement and a separate calvarial lesion with dural thickening and enhancement that was concerning for an additional meningioma, consistent with the multifocal potential described in RIM cohorts.

Latency from irradiation to meningioma diagnosis is typically reported in decades, with median values frequently in the 20-30-year range in large cohorts. However, substantial variability exists, and shorter latency has been observed, particularly in patients irradiated at younger ages [3,12,16]. A dose-dependent increase in risk has been reported in survivor studies, supporting a dose-response relationship for secondary meningiomas following pediatric cranial irradiation [1,4]. Our patient developed his initial sphenoid wing meningioma approximately 13 years after cranial irradiation for medulloblastoma at age three, representing an earlier-than-typical presentation and aligning with the concept that very young age at exposure may be associated with shorter latency.

Skull base and sphenoid wing RIMs pose added complexity because of their proximity to the optic apparatus, cavernous sinus, and major vascular structures. Orbital and sinonasal extension can also contribute to compressive symptoms and complicate surgical corridors. Although multiple studies characterize RIM behavior broadly, detailed location-specific outcomes for skull base lesions, particularly sphenoid wing tumors with orbital and paranasal sinus involvement, remain incompletely described. This limits evidence-based guidance on resection feasibility, recurrence risk, and follow-up strategies in this subgroup. In practice, operative planning for recurrent sphenoid wing lesions with orbital extension may require individualized skull base exposure, including frontotemporal or orbitozygomatic-type corridors depending on disease extent, while balancing maximal safe resection against risk to adjacent visual and neurovascular structures [19]. In our patient, progressive regrowth involved the orbit, frontal sinus, and ethmoid air cells, with associated proptosis and visual symptoms, illustrating the anatomic and symptomatic burden these lesions can impose.

Surveillance recommendations for childhood cancer survivors treated with cranial radiotherapy remain variable, with proposed MRI screening strategies differing by cohort, risk stratification, and local practice patterns. Several reports suggest that risk emerges within the first decade after radiation and continues to rise for decades thereafter, supporting long-term monitoring. However, the optimal imaging interval for asymptomatic survivors remains uncertain [3,6,18]. This case highlights the clinical relevance of sustained surveillance. A small lesion identified at age 25 progressed over three years to multifocal symptomatic disease with orbital involvement, underscoring the potential for clinically meaningful progression between imaging intervals.

Limitations of this report include the unavailability of details regarding the original radiation dose and treatment field, which limits patient-specific dose-response interpretation. Earlier imaging and operative records, including imaging prior to 2022 and the initial pathology report from the prior meningioma resection, were not available, restricting longitudinal assessment of tumor evolution, growth kinetics, and histologic comparison over time. In addition, histopathology for the current recurrent lesions was not available at the time of writing, precluding comparison with reported WHO grade distributions and molecular features. The absence of molecular profiling further limited comparison with reported radiation-associated genomic alterations and distinction from sporadic molecular patterns. Follow-up is limited by deferred definitive management and the potential for loss to follow-up.

## Conclusions

RIMs are an important late effect of pediatric cranial radiotherapy and can present with multifocal disease, recurrence, and clinically meaningful progression over time. Our case illustrates the management complexity of recurrent skull base disease, with sphenoid wing involvement and extension into the orbit and paranasal sinuses, where complete resection may be limited by adjacent critical neurovascular and visual structures. Because risk persists for decades after childhood irradiation and screening practices vary, long-term risk-informed surveillance remains essential. Further studies describing location-specific outcomes for skull base RIMs are needed to better inform operative planning and follow-up strategies.
